# Domestic Summary

**Published:** 1839-07-06

**Authors:** 


### DOMESTIC SUMMARY.

*Medical College of Philadelphia.*—The bill incorporating this institution has passed the legislature of Pennsylvania.

